# Application of Sparse Representation to Bartlett Spectra for Improved Direction of Arrival Estimation

**DOI:** 10.3390/s21010077

**Published:** 2020-12-25

**Authors:** Jacob Compaleo, Inder J. Gupta

**Affiliations:** ElectroScience Laboratory, The Ohio State University, Columbus, OH 43212, USA; Compaleo.2@osu.edu

**Keywords:** direction of arrival (DOA) estimation, sparse representation, Bartlett spectra

## Abstract

A new technique for high-resolution direction of arrival estimation is presented. The method utilizes the traditional Bartlett spectra and sparse representation to locate emitters in single and multiple emitter scenarios. A method for selecting the sparse representation regularization parameter is also presented. Using Monte Carlo simulations, we show that the proposed approach achieves accurate direction of arrival (DOA) estimations that are unbiased and a variance that approaches the Cramer–Rao lower bound. We show that our method outperforms the popular MUSIC algorithm, and is slightly better than the sparse representation based L1-SVD algorithm when angular separation between emitters is small, signal SNR is low, and a small number of snapshots are used in DOA estimation.

## 1. Introduction

Since World War II, obtaining accurate direction of arrival (DOA) estimation of the signals incident on an array of sensors has been an area of great interest. One of the earliest formulated DOA estimation methods that is still widely used today is Bartlett’s method [1]. Bartlett’s method has remained popular due to its simplicity and straight-forward approach. For single emitter scenarios, Bartlett’s method is successful in estimating the DOA. In fact, for the single emitter case in the presence of white Gaussian noise, it is the maximum likelihood solution. For the case with multiple emitters in a single scene, the failure rate of Bartlett’s method increases as these emitters become more closely spaced together [2]. As Bartlett’s method is a biased estimator in scenarios with multiple emitters, it is preferred to use other higher resolution, unbiased algorithms.

Multiple signal classification (MUSIC) [3] algorithm is another widely used DOA estimation algorithm. MUSIC, as its name suggests, provides a method to estimate the DOA of multiple emitters in a single scene at a high resolution and large success rate. Despite MUSIC’s advantages over Bartlett’s method, it is not without caveats of its own. In scenarios with a low number of snapshots being utilized, as well as in cases with low angular separation between emitters, MUSIC’s performance begins to suffer and the algorithm fails at resolving separate signals [4].

The concept of sparse representation has found a plethora of applications such as image classification [5], computer vision [6], and machine learning [7]. The minimization capabilities of sparse representation have also made it a popular tool for DOA estimation. Many examples of sparse representation applications for DOA estimation can be found in the literature. In [8,9,10,11,12,13,14,15,16,17,18,19], sparse representation is applied in the data domain, often to the covariance matrix. Another popular approach to the sparse representation DOA estimation problem is to use Bayesian Learning [20,21,22,23]. Recently, much work on the topic of sparse arrays, such as co-prime or nested arrays, has been proposed as a solution for the DOA estimation problem [24,25,26,27,28,29,30]. We propose a new approach that utilizes sparse representation for DOA estimation. In the proposed approach, sparse representation is applied in the spectral domain. Bartlett’s method is first used to obtain the DOA estimate in the spectral domain. Despite its issues with closely-spaced emitters, the Bartlett spectra holds a lot of information that can be exploited with sparse representation. Our approach is based on the assumption that the Bartlett spectra computed using the received signals is a superposition of the Bartlett spectra of individual RF emitters. The superposition assumption allows us to create a dictionary matrix of Bartlett spectra. We can then compare a Bartlett spectrum of interest with this dictionary matrix using sparse representation to achieve accurate DOA estimation.

The first step for the algorithm requires generating the Bartlett spectra for a given scene. From here, we focus solely on regions of interest, the major lobes of the Bartlett spectrum. Sources are populated within the major lobes of the Bartlett spectrum. A single emitter Bartlett spectra is generated for each source. Each single emitter Bartlett spectra becomes a column of a dictionary matrix used for sparse representation. Next, the sparse representation constraint is applied to solve for the solution vector that contains the DOA estimates. One noted caveat of using sparse representation is the need for a regularization parameter that constrains the solution [31]. Various methods for estimating or computing this regularization parameter have been published [32,33]. Unfortunately, these methods are not without their own impediments. For our algorithm, we build on the method presented in [34] and bolster it into an automated approach.

We presented the basic concept behind the proposed method in a conference paper [35] in 2019. In the paper, analytical results based on infinite number of snapshots for a single RF scenario were discussed. Also, at that time the method to select the regularization parameter was not fully developed. In this paper, we present our approach to select the sparse parameter and a thorough statistical performance of the proposed method using Monte Carlo simulations.

In this paper, using Monte Carlo simulations, we show that the proposed method leads to unbiased DOA estimates and is statistically efficient in that the variance in the estimated DOA approaches the Cramer–Rao bound. We also demonstrate that for small angular separation between the incident signals, the proposed method outperforms the popular MUSIC algorithm for DOA estimation, as well as the conventional sparse representation method that is applied in the data domain [33]. This is especially true when SNR of the incident signals is low and a few snapshots of the incident signals are used in DOA estimation.

The remainder of this paper is organized in the following way. Section 2 discusses the basic signal model setup and the formulation of Bartlett’s method for DOA estimation. Section 3 expounds our proposed method of applying sparse representation to the Bartlett spectra. Section 4 presents our method of selecting the sparse representation regularization parameter. Section 5 shows Monte Carlo simulation results comparing our method, MUSIC, L1-SVD, and the Cramer–Rao bound for a two-signal scenario over varying angular separation, SNR, and snapshot cases. Lastly, in Section 6 we discuss our results and provide a conclusion.

## 2. Signal Model and Bartlett’s Method

Let *K* overlapping narrowband signals be received by an *N* element antenna array. For this work, overlapping means the signals are received at the same frequency and time. The received signal at the *i*th element can be represented as [36]

(1) $$y_{i}\left(t\right)=\left(\sum_{k=1}^{K}d_{i}\left(\phi_{k}\right)s_{k}\left(t\right)\right)+\nu_{i}\left(t\right)$$

where $s_{k}\left(t\right)$ is the $k^{th}$ signal received by an isotropic antenna located at the coordinate origin (the center of the antenna array), $d_{i}\left(\phi_{k}\right)$ is the gain and phase shift of the *i*th element of the antenna in the $k^{th}$ emitter direction, $\phi_{k}$, and $\nu_{i}\left(t\right)$ is the thermal noise. Note that $d_{i}\left(\phi_{k}\right)$ includes the phase shift due to the element being not at the coordinate origin. We assume the noise to be uncorrelated with the incident signals, and also between the various antenna elements. Further noise is assumed to be complex circular Gaussian with unity variance. For all *N* elements, (1) can be written in vector form as

(2) $$y\left(t\right)=\left(\sum_{k=1}^{K}d\left(\phi_{k}\right)s_{k}\left(t\right)\right)+\nu \left(t\right)$$

where y(t) is the received signal vector of length *N*, $d(\phi_{k})$ is referred to as the antenna array manifold vector of length *N*, and ν(t) is the noise vector of length *N*. The equation can be represented in matrix form to remove the summation as

(3) y(t)=Ds(t)+ν(t)

where

$$D=[d\left(\phi_{1}\right),\ldots ,d\left(\phi_{K}\right)]$$

$$s\left(t\right)={\left[s_{1}\left(t\right),...,s_{K}\left(t\right)\right]}^{T}$$

D is a matrix of size *N* by *K*, s(t) is a vector of length *K*. The received signals are downconverted and digitized using an analog to digital converter (ADC). Let the signal be digitized with a sampling period of *T* seconds. The *l*th sample after digitization is represented as

(4) y[l]=Ds[l]+ν[l]

and (lT) is written concisely as [l]. y[l] is referred to as the snapshot vector. Let *P* snapshots be used to estimate the DOA, the snapshot vector can be represented in matrix form as

(5) Y=[y[1],…,y[P]]

where Y is now referred to as the snapshot matrix.

A popular DOA estimation technique, Bartlett’s method, uses the antenna array manifold and snapshot matrix to estimate the signal direction. The equation for the Bartlett spectrum along a direction ϕ can be written as

(6) $$b(\phi )=\frac{{d(\phi )}^{H}\hat{R}d(\phi )}{{d(\phi )}^{H}d(\phi )}$$

where d(ϕ) is the antenna array manifold in the direction ϕ and $\hat{R}$ is the sample covariance matrix. The sample covariance matrix is represented as

(7) $$\hat{R}=\frac{1}{P}YY^{H}$$

where *H* is the Hermitian transpose. By varying ϕ, one can calculate the Bartlett spectrum over the angular region of interest. Then, the peaks in the Bartlett spectra correspond to the directions of the incident signals.

**Figure 1:**
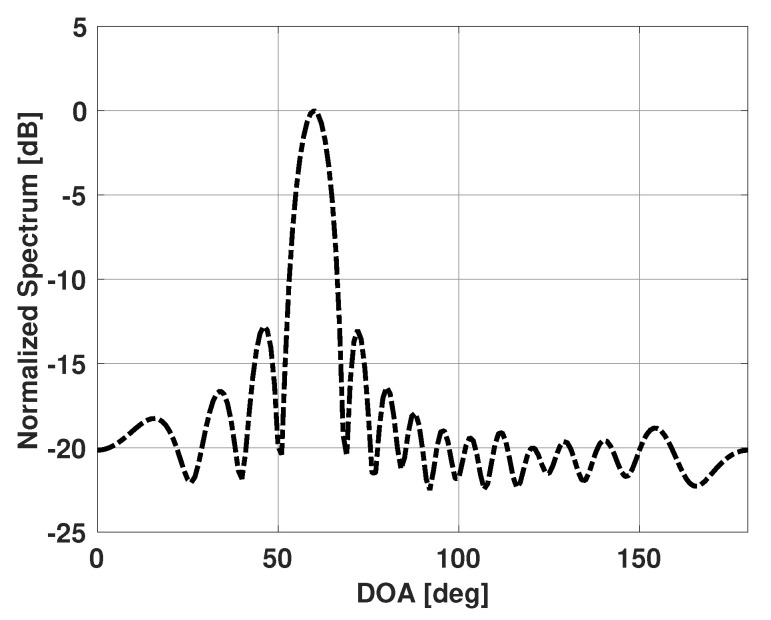
Bartlett spectrum versus direction of arrival (DOA) for a uniform linear array of 15 elements in the presence of a single signal incident at 60°.

Figure 1 shows the Bartlett spectra for a linear uniform antenna array of fifteen isotropic elements spaced half a wavelength apart in the presence of a single signal incident at 60° with respect to the antenna array end fire direction. The signal has an SNR of 10 dB and 100 snapshots are used to obtain the Bartlett spectra. Note that there is one major lobe present in the spectrum and the peak corresponds to the incident signal direction. The Bartlett method, therefore, is able to successfully estimate DOA for scenarios with a single incident signal.

**Figure 2:**
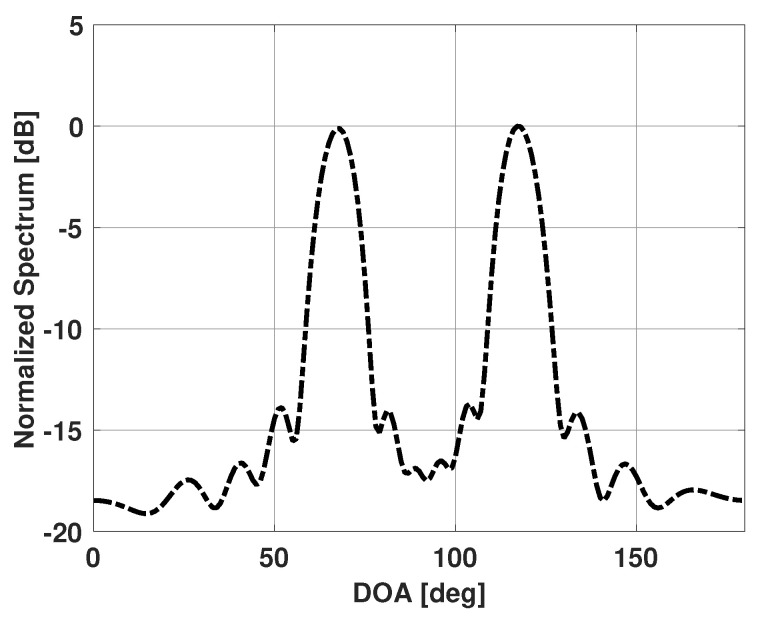
Bartlett spectrum versus DOA for a uniform linear array of 15 elements in the presence of four signals incident at 65°, 70°, 115°, and 120°.

Figure 2 shows the Bartlett spectra when four signals are incident on the antenna array. The signals are incident at 65°, 70°, 115°, and 120°. Each signal has an SNR of 10 dB and 100 snapshots are used to obtain the Bartlett spectra. Note that now only two major lobes are apparent in the spectra despite there being 4 incident signals. The major lobes of the signals spaced closely together have merged, and the peaks now correspond to a direction in-between the closely-spaced signals. Bartlett method is therefore failing to estimate the DOA with multiple signals spaced closely together. In the next section, we present our proposed solution to improve this DOA estimation problem with multiple incident signals. In the proposed method, we will use the Bartlett spectra to improve DOA estimation. The spectra for all values of ϕ will be written as a vector b.

## 3. Proposed Method

**Figure 3:**
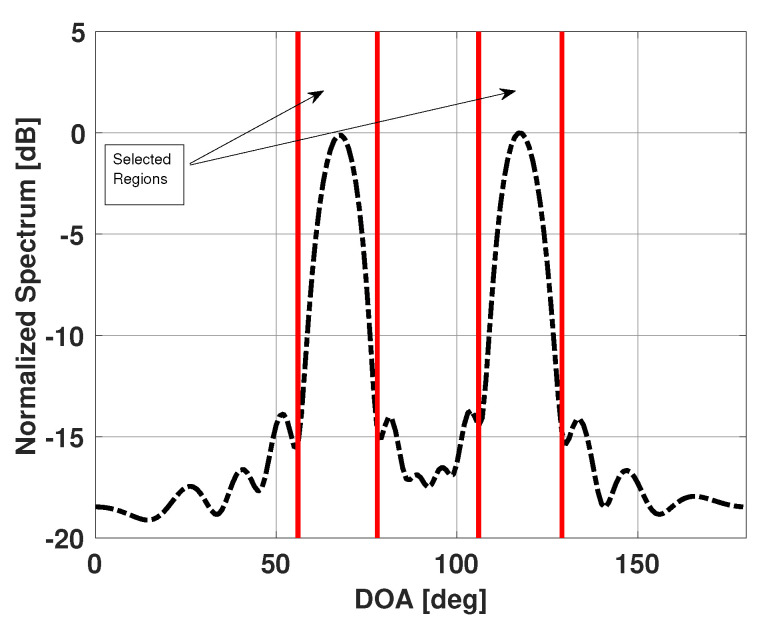
Angular regions selected from the Bartlett spectra for a uniform linear array of 15 elements in the presence of four signals incident at 65°, 70°, 115°, and 120°.

Our method is based on the assumption that the observed Bartlett spectra is a superposition of the Bartlett spectra of a few individual emitters present in the scene plus some noise. The directions of these emitters and their signal strengths (incident power) at the coordinate origin however, are unknown. One can use the observed Bartlett spectra, b, to obtain the possible angular regions that contain the unknown emitters. The observed Bartlett spectra will have high values in those angular regions. For example, from the observed Bartlett spectra shown in Figure 2, one can say that the angular regions around 67° and 117° contain the emitters in the scene. Figure 3 shows the possible angular regions of interest.

In our approach, one populates these angular regions of interest with many closely spaced (say every 0.1°) emitters and calculates the individual Bartlett spectra for each of these emitters assuming that each emitter has signal strength of unity. These Bartlett spectra are calculated along the same directions as the observed Bartlett spectra, b. Note that one can use the available antenna array manifold to calculate the individual Bartlett spectra. For a unit power emitter located along direction $\phi_{m}$, the Bartlett spectra in direction ϕ is given by

(8) $$a_{m}\left(\phi \right)=\frac{|d^{H}\left(\phi \right)d\left(\phi_{m}\right){|}^{2}}{\left(d^{H}\left(\phi \right)d\left(\phi \right)\right)}$$

m=1,2,…,M

where *M* is the total number of emitters distributed in the angular regions of interest. These *M* Bartlett spectra form the dictionary of interest. We want to select a few elements of this dictionary to represent the observed Bartlett spectra, b. This is a sparse problem.

Let vector x of length *M* represent the signal strength of the individual emitters that will lead to the desired match between the observed spectra and the combined spectra of the individual emitters. Then, one can find x by solving the following well-known optimization problem [37]

(9) $$min{\left\|x(\beta )\right\|}_{0}$$

such that

(10) $${\left\|Ax(\beta )-b\right\|}_{2}^{2}\leq \beta {\left\|b\right\|}_{2}^{2}$$

where A is a matrix of *M* columns with each column representing the Bartlett spectra of an individual emitter of signal strength unity as computed in (8), b is the observed Bartlett spectra, and β is the sparse representation regularization parameter. In (9), ${\left\|.\right\|}_{0}$ represents the 0th order norm of a vector. By minimizing ${\left\|x(\beta )\right\|}_{0}$, we are minimizing the number of non-zero elements of x(β), which is therefore a sparse solution. In (10), ${\left\|.\right\|}_{2}^{2}$ is the square of the Euclidean norm, also referred to as the L2-norm. The sparse solution of x depends on the selected value of β and that is the reason we have selected to write x as a function of β; i.e., x(β).

Unfortunately, (9) and (10) is an optimization problem that is very difficult to solve. It has been shown that this L0-norm minimization can be relaxed to the L1-norm minimization [37]. This L1-norm minimization problem is solved as [33]

(11) $$min{\left\|x(\beta )\right\|}_{1}$$

such that

(12) $${\left\|Ax(\beta )-b\right\|}_{2}^{2}\leq \beta {\left\|b\right\|}_{2}^{2}$$

**Figure 4:**
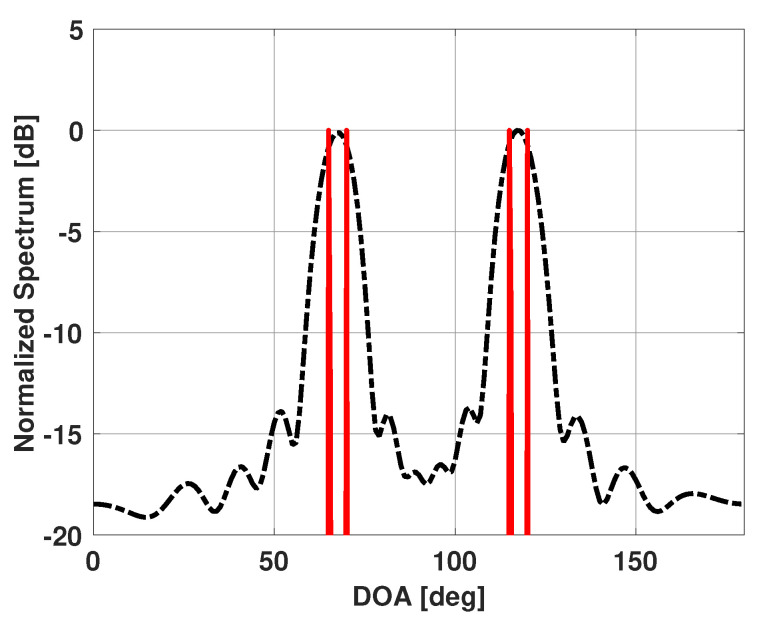
Solution vector x versus DOA for a uniform linear array of 15 elements in the presence of four signals at 65°, 70°, 115°, and 120°.

As we are dealing with power, we can add the additional constraint that all entries of vector x(β) must be positive. The L1-norm minimization has been shown to have advantages of its own [38]. Namely, L1 minimization favors sparse values of x, and also is less computationally expensive compared to other norm minimizations. In the present work, we used the MATLAB optimization package SeDuMi [39] to solve the constraint minimization problem. Figure 4 shows a plot of solution vector x(β) for the previous example of four incident RF emitters. Note that the plot contains four peaks in the directions of the four incident emitters.

In the above optimization problem, β sets the amount of mismatch that one is willing to allow to get a good sparse solution. Note that β>0 and should be selected carefully. Our approach to select β is discussed in the next section.

## 4. Regularization Parameter Selection

From Equations (11) and (12), the range of potential β values can be acknowledged as 0<β<1. Note that when β=0, our forward model perfectly matches the observations (vector b). When β=1, the obvious solution for x is a null vector due to the constraint that all entries of x must be positive, and all elements of matrix A and vector b are real positive numbers. Also, from (11) and (12), one can conclude that as β increases, the norm of vector x will continuously decrease. Let $x_{0}$ be the least squares solution of Ax=b, and

(13) $$\beta_{0}=\frac{{\left\|Ax_{0}-b\right\|}_{2}^{2}}{{\left\|b\right\|}_{2}^{2}}$$

**Figure 5:**
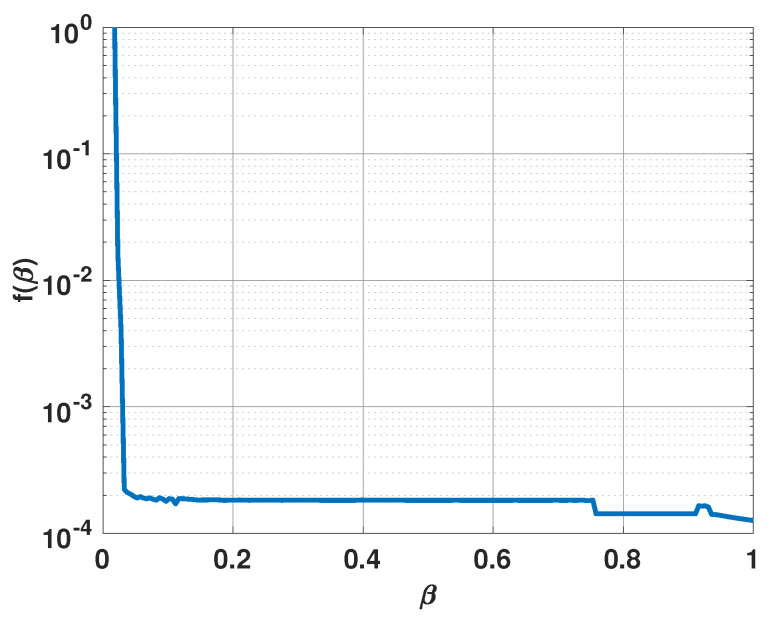
f(β) versus β for a uniform linear array of 15 elements in the presence of three signals at 55°, 75°, and 95°.

Then, $\beta_{0}$ is the smallest possible value of β. For an accurate solution, we want β as close to $\beta_{0}$ as possible. If β is too small, it is known that spurious peaks will be present in the solution vector [33]. If β is too large, signal peaks may merge together and an accurate DOA estimate is no longer attainable. To find this value of β, we investigated the rate of change in ${\left\|x(\beta )\right\|}_{1}$ versus β. Mathematically, we investigated f(β) defined as

(14) $$f\left(\beta \right)=\frac{{\left\|x(\beta )-x(\beta +\Delta )\right\|}_{1}}{{\left\|x_{0}\right\|}_{1}}$$

where Δ represents an incrementing variation of β. We found that initially, f(β) decreases rapidly with an increase in β. Once β reaches a certain value, f(β) no longer sees a rapid rate of change with an increase in β. We found that the smallest value of β after which f(β) does not change much is a good choice for β. For example, Figure 5 shows a plot of f(β) versus β for a linear uniform antenna array of fifteen isotropic elements spaced half a wavelength apart in the presence of three signals. Each signal has an SNR of 5 dB and 100 snapshots are used to obtain the Bartlett spectra. All signals are incident in the same plane and the angle of arrival of the three signals are 55°, 75°, and 95° with respect to the antenna array end fire direction. Note that in the figure, f(β) has a clear inflection point. We propose to use the smallest value of β that leads to this inflection point as the β value for our approach.

At the inflection point, the derivative of f(β) with respect to β will be very small (close to zero). We propose to use the smallest value of β at which the derivative of f(β) is less than or equal to $10^{-5}$, i.e.,

(15) $$\frac{d}{d\beta }f(\beta )\leq 10^{-5}$$

**Figure 6:**
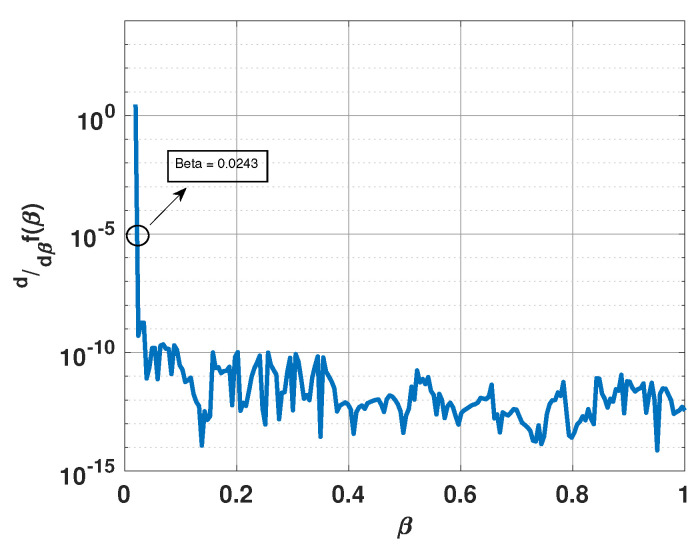
Derivative of f(β) versus β for a uniform linear array of 15 elements in the presence of three signals at 55°, 75°, and 95°.

**Figure 7:**
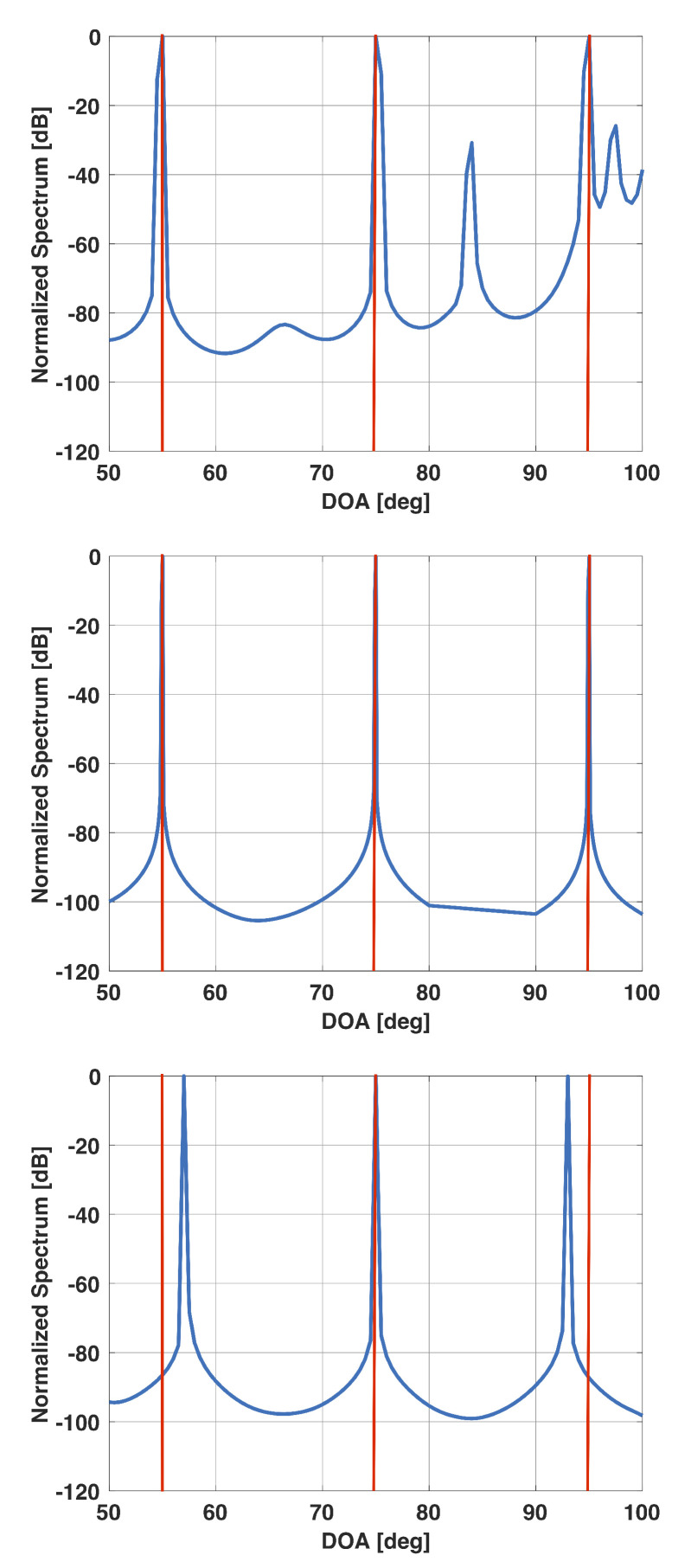
Solution vector x versus DOA for a uniform linear array of 15 elements in the presence of three signals at 55°, 75°, and 95°. For this scenario, $\beta_{0}=0.0171$. β=0.02 (**top**), β=0.0243 (selected beta) (**middle**), β=0.1 (**bottom**).

Figure 6 shows a plot of the derivative of f(β) versus β for the above example. From this plot, it is clear that the derivative becomes small for β>0.03 and is equal to $10^{-5}$ for β=0.0243. Figure 7 shows a plot of x for this value of β. Note that the plot has sharp peaks along the directions of the three incident signals. For this example, $\beta_{0}=0.0171$. In Figure 7, we also include a plot of x when β is selected to be 0.02 and 0.1. Note that for β=0.02, x has more peaks than the number of incident signals; whereas for β=0.1, the peaks are not at the right locations.

**Figure 8:**
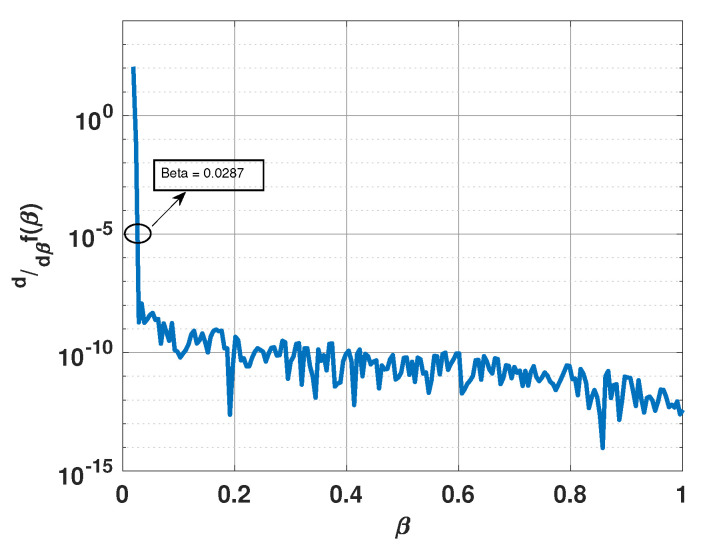
Derivative of f(β) versus β for a uniform linear array of 15 elements in the presence of three signals at 57°, 62°, and 67°.

**Figure 9:**
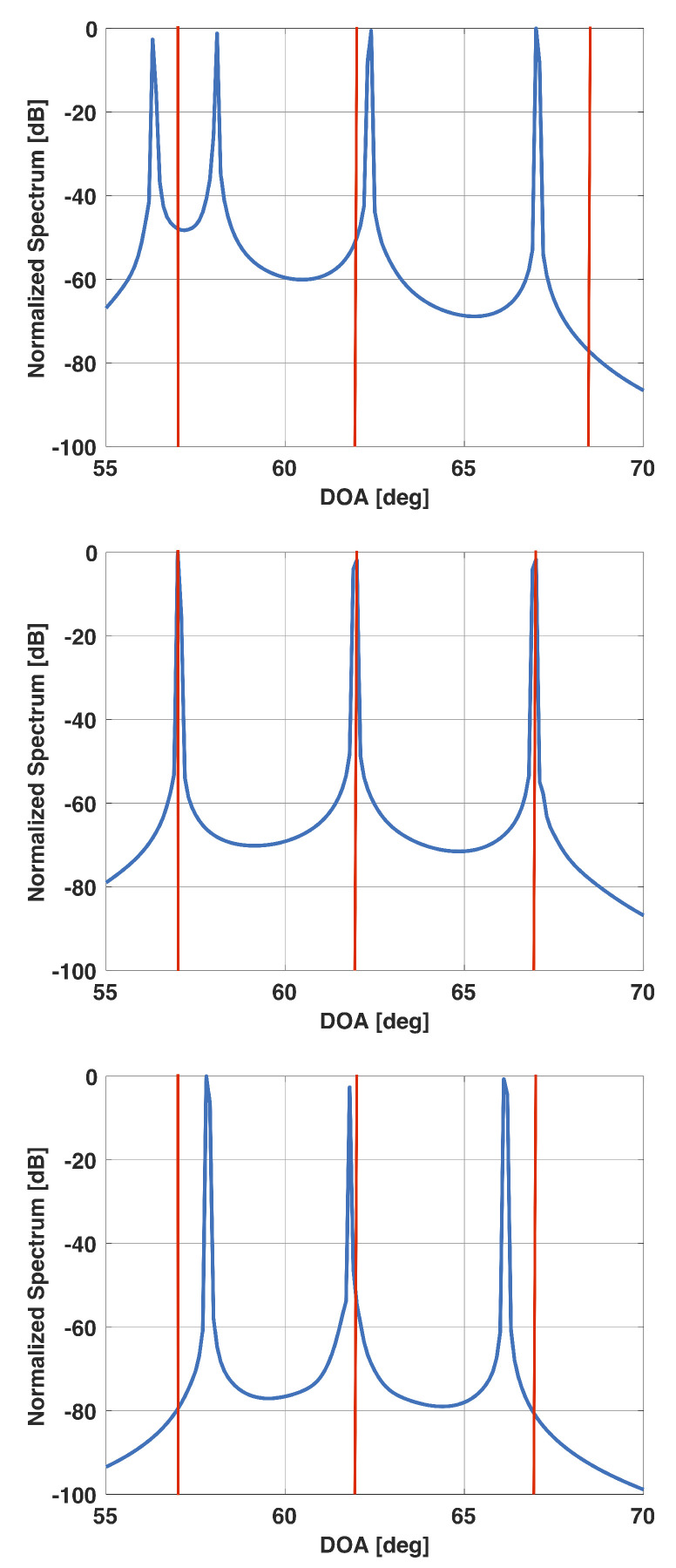
Solution vector x versus DOA for a uniform linear array of 15 elements in the presence of three signals at 57°, 62°, and 67°. For this scenario, $\beta_{0}=0.0185$. β=0.02 (**top**), β=0.0287 (selected beta) (**middle**), β=0.1 (**bottom**).

Figure 8 shows a plot of the derivative of f(β) versus β when the three signals are incident from 57°, 62°, and 67°, respectively. All other parameters are the same as before. Again, one can observe the same trend. For this scenario, the derivative approaches $10^{-5}$ for β=0.0287. Figure 9 shows a plot of x for this value of β. Note that the plot has sharp peaks along the directions of the three incident signals. For this example, $\beta_{0}=0.0185$. In Figure 9, we also include a plot of x when β is selected to be 0.02 and 0.1. Note that for β=0.02, x again has more peaks than the number of incident signals; whereas for β=0.1, the peaks are not at the right locations.

## 5. Results

We performed Monte Carlo simulations to evaluate the performance of the proposed method for high resolution DOA estimates. For each signal scenario, 500 independent trials were carried out. Results of these trials were used to calculate the bias and variance in the estimated DOA. The variance data was compared with the Cramer–Rao Bound (CRB) [40]. We used a uniform linear antenna array of fifteen isotropic antenna elements in the simulations. The antenna elements were placed along the x-axis and had an interelement spacing of half a wavelength. All the signals were incident in the same plane that also contained the antenna array and were assumed to be of the same strength (equal SNR). The angle of arrival was measured with respect to x-axis. Thus, 90° represents broadside to the antenna array.

In the results presented in this paper, two narrowband signals are incident on the antenna array. One of the signals is incident from 60°; whereas the direction of the other signal, unless mentioned, is varied. Along 60°, the null-to-null beamwidth of the antenna array is approximately 18°. Thus, Bartlett method will not be able to resolve signals that have angular separation of less than 9 to 10 degrees, leading to large bias and variance in the estimated signal directions. When the signals have large angular separations, separate peaks can be resolved, though estimated directions can still be biased.

**Figure 10:**
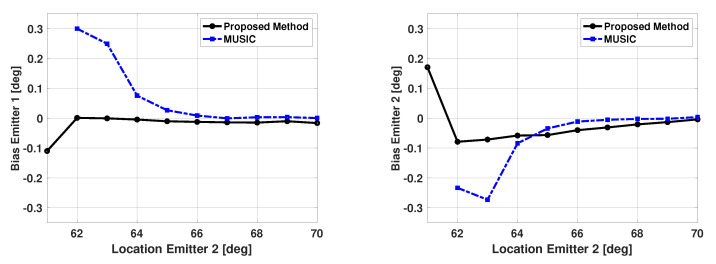
Biases in the estimated direction of the two emitters versus angular separation: Proposed Method and the MUSIC Algorithm, SNR = 5 dB, 100 snapshots, 500 trials.

**Figure 11:**
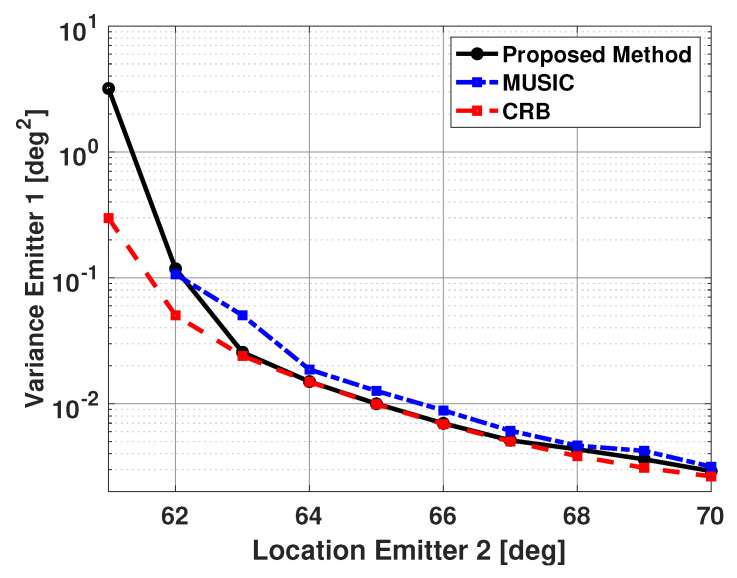
Variance in the estimated direction of the first emitter versus angular separation: Proposed Method and the MUSIC Algorithm, SNR = 5 dB, 100 snapshots, 500 trials.

Figure 10 and Figure 11 show the bias and variance in the estimated signal directions when the proposed method is used for DOA estimation. The two signals have a SNR of 5 dB at each antenna element and 100 snapshots are used to estimate the signal directions. The incident angle of the second signal is varied from 61° to 70°, and biases and variance are plotted versus the second signal direction. The variance in the estimated directions of the two signals was very similar. Therefore, in all the results presented in this paper, the variance results for only one of the signals are shown. Also, in all these results, vector b is a size 181x1 Bartlett spectra that spans from 0 to 180 degrees with a step size of 1 degree. In Figure 10 and Figure 11, one can note the proposed method is unbiased and efficient. Note the variance of the proposed method is reaching CRB. For comparison purposes, the statistics of the results obtained using the popular MUSIC algorithm are also included in these figures. For the MUSIC algorithm, the signal subspace dimension is selected to be two, which is equal to the number of incident signals. From the figures, one can note that for small angular separation between the two signals, the proposed method is outperforming the MUSIC algorithm.

The results shown in these figures are based on the successful trials only. We define a trial to be successful if one obtains individual peaks along each incident signal direction. Table 1 shows the number of failures versus the incident angle of the second signal for the proposed method and the MUSIC algorithm. Note that the MUSIC algorithm has significantly more failures than the proposed algorithm. The bias and variance for the MUSIC algorithm are not computed for angular separation of 1 degree. This is because all 500 trials for MUSIC were unsuccessful for this angular separation. Also, from the plots in Figure 10 and Figure 11, one can see that even for successful trials, the proposed algorithm has better performance than the MUSIC algorithm.

As previously mentioned, regularization parameter β is a critical component to the use of sparse representation. Table 2 shows the average minimum β value, $\beta_{0}$, as well as the average selected β value over 500 trials at each emitter 2 location. From the table, one can see that the selected value of β is very close to $\beta_{0}$.

**Figure 12:**
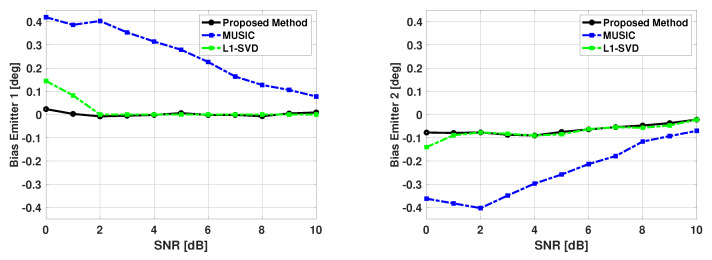
Biases in the estimated direction of the two emitters versus SNR: Proposed Method, MUSIC Algorithm, and L1-SVD Algorithm, Emitters at 60 and 63 deg, 100 snapshots, 500 trials.

**Figure 13:**
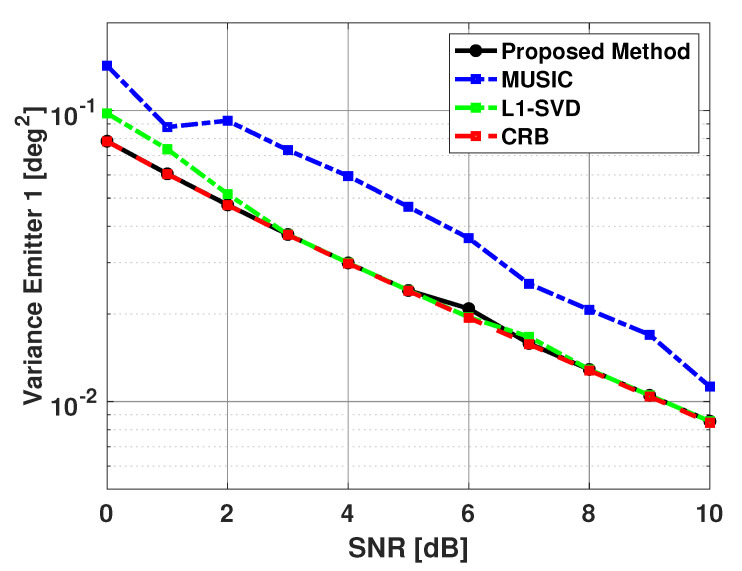
Variance in the estimated direction of the first emitter versus SNR: Proposed Method, MUSIC Algorithm, and L1-SVD Algorithm, Emitters at 60 and 63 deg, 100 snapshots, 500 trials.

From Table 1, we observed that MUSIC experiences a significant number of failures when angular separation is 3 degrees or less. It is known that increasing SNR or increasing the number of snapshots improves DOA estimation performance. Figure 12 and Figure 13 show the bias and variance in the estimated signal directions when SNR is varied from 0 to 10 dB. The angle of arrival of the two signals are fixed at 60 and 63 degrees, and 100 snapshots are used in DOA estimation. Again, b is a size 181x1 Bartlett spectra that spans from 0 to 180 degrees with a step size of 1 degree. One can again note from Figure 12 and Figure 13, the proposed method is unbiased and efficient.

For the simulation results shown in Figure 12 and Figure 13, we chose to compare our proposed method with another sparse representation based DOA estimation technique, as well with the MUSIC algorithm. The L1-SVD algorithm is the conventional DOA estimation approach when applying sparse representation in the data domain [33]. The L1-SVD algorithm focuses on recovering a sparse signal vector using received array data and an over-complete basis of potential matches to the contributions of each signal. This recovery is based on the assumption that the signal subspace is sparse. As the algorithm name implies, sparse representation is applied in an L1-norm sense to the singular value decomposition (SVD) of the received array data to recover the sparse signal vector. This SVD can be thought of as very similar to the eigen-decomposition of the covariance matrix of the received array data. For a complete discussion of the L1-SVD algorithm, please refer to [33]. The L1-SVD algorithm has its own sparse representation regularization parameter selection method. The L1-SVD regularization parameter selection method relies on the assumption that knowledge of the noise characteristics is readily available. While this is not always feasible, the method will be used to generate the L1-SVD DOA estimates for these results. To be consistent with the proposed method, L1-SVD algorithm is simulated with the same grid of potential emitter locations for these results. From Figure 12 and Figure 13, one can see that the proposed method has better performance than the MUSIC algorithm for all SNR values shown, and a little better performance than the L1-SVD algorithm when SNR is low.

The results shown in Figure 12 and Figure 13 are computed using successful trials only. Table 3 shows the number of failures versus SNR for the signals. The MUSIC algorithm is shown to have a large amount of failures at low SNR values. Thus, one can claim that the proposed method has much better performance than the MUSIC algorithm. The proposed method and the L1-SVD algorithm have no failures. However, based on the bias and variance plots shown in the last two figures, we can say that the proposed method has slightly better performance than the L1-SVD algorithm.

Table 4 shows the average $\beta_{0}$ and selected β value over 500 trials at each SNR value. Note that, as expected, $\beta_{0}$ decreases with an increase in the SNR of the incident signals and selected β is close to $\beta_{0}$.

**Figure 14:**
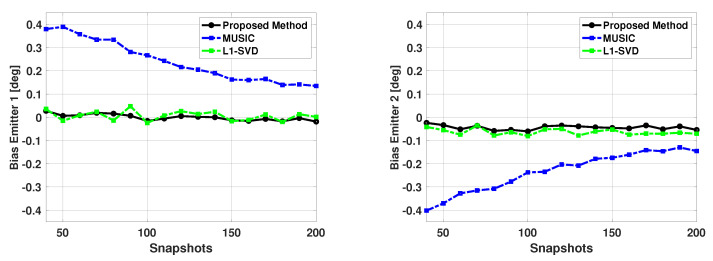
Biases in the estimated direction of the two emitters versus snapshots: Proposed Method, MUSIC Algorithm, and L1-SVD Algorithm, Emitters at 60 and 63 deg, SNR = 5 dB, 500 trials.

**Figure 15:**
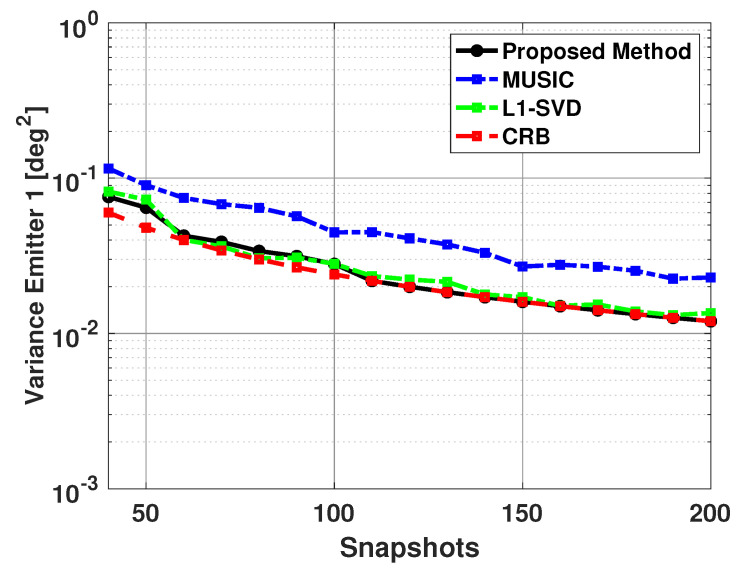
Variance in the estimated direction of the first emitter versus snapshots: Proposed Method, MUSIC Algorithm, and L1-SVD Algorithm, Emitters at 60 and 63 deg, SNR = 5 dB, 500 trials.

Next we fixed the SNR of the two emitters at 5 dB, and kept the signal directions at 60 and 63 degrees, but varied the number of snapshots from 40 to 200. Again, b is a size 181x1 Bartlett spectra that spans from 0 to 180 degrees with a step size of 1 degree. Figure 14 and Figure 15 show the bias and variance versus the number of snapshots used in DOA estimation. Again, only successful trials are used to compute the bias and variance. From Figure 14 and Figure 15, one can note the proposed algorithm is unbiased and efficient and has much better performance than the MUSIC algorithm. Also, it can be noted that while the proposed method and L1-SVD algorithm have similar variance, the bias of the proposed method is more consistently at zero and has less fluctuations than the L1-SVD algorithm.

Table 5 shows the number of failures versus the number of snapshots used in DOA estimation. Note that when using a low number of snapshots, the MUSIC algorithm is not able to resolve the directions of the two signals. In addition to this, the L1-SVD algorithm also has a few failures when a small number of snapshots are used in DOA estimation. The proposed method is able to resolve the directions of the two signals for all values of the number of snapshots used in DOA estimation. Thus, the proposed method outperforms the MUSIC algorithm and L1-SVD algorithm, especially when a small number of snapshots is used in DOA estimation.

Table 6 shows the average $\beta_{0}$ and selected β value over 500 trials versus the number of snapshots used in the DOA estimation. Note that $\beta_{0}$, as expected, is almost invariant with the number of snapshots used in the DOA estimation. Also, selected β is close to $\beta_{0}$. From the above results, we can conclude that the proposed method is very successful for DOA estimation.

## 6. Summary and Conclusions

We have presented a new approach for the DOA estimation of multiple overlapping emitters incident on a sensor array. The approach is based on applying sparse representation to the Bartlett spectra obtained from the snapshots of the signals received by various array elements. We also introduced a method for the automatic selection of the regularization parameter used in sparse representation. Using Monte Carlo simulations, we have shown that the proposed approach leads to unbiased DOA estimates and is statistically efficient. We compared the performance of the proposed method with that of the MUSIC algorithm, as well as the conventional application of sparse representation in the data domain [33]. It was shown that for small angular separations between the emitters, the proposed approach has better performance than the MUSIC algorithm. The proposed method also performs slightly better than a L1-SVD based algorithm investigated in this paper. This is especially true when the incident signals have low SNR. In the proposed approach, the angular region of interest is reduced to the major lobes of the Bartlett spectra. Thus, the proposed approach has low computational complexity.

In the results presented in this paper, the two incident signals are assumed to have similar SNR. In practice, this will be hardly true. The proposed method works as well in the presence of signals with unequal signal strength. For a large dynamic range of the incident signals, one is better off using a non-uniform window function in the generation of the Bartlett spectra. The selection of the window function will be discussed in a future paper that is under preparation.

## Figures and Tables

**Table 1:** Number of Failures in 500 Trials with Respect to Emitter 2 Location. SNR = 5 dB, 100 snapshots.

Location Emitter 2 [deg]	MUSIC	Proposed Method
61	500	13
62	497	0
63	30	0
64	2	0
65	0	0

**Table 2:** Average $\beta_{0}$ and selected β value over 500 Trials with Respect to Emitter 2 Location. SNR = 5 dB, 100 snapshots.

Location Emitter 2 [deg]	$\beta_{0}$	β
61	0.0491	0.0638
62	0.0517	0.0670
63	0.0518	0.0674
64	0.0570	0.0743
65	0.0569	0.0740
66	0.0592	0.0769
67	0.0624	0.0808
68	0.0640	0.0830
69	0.0659	0.0852
70	0.0681	0.0878

**Table 3:** Number of Failures in 500 Trials with Respect to SNR. Two emitters at 60° and 63°, 100 snapshots.

SNR [dB]	MUSIC	L1-SVD	Proposed Method
0	473	0	0
1	396	0	0
2	338	0	0
3	221	0	0
4	118	0	0
5	44	0	0
6	6	0	0
7	3	0	0
8	3	0	0
9	2	0	0
10	2	0	0

**Table 4:** Average $\beta_{0}$ and selected β value over 500 Trials with Respect to SNR. Two emitters at 60° and 63°, 100 snapshots.

SNR [dB]	$\beta_{0}$	β
0	0.1543	0.2006
1	0.1287	0.1672
2	0.1002	0.1304
3	0.0834	0.1084
4	0.0651	0.0847
5	0.0518	0.0674
6	0.0427	0.0558
7	0.0334	0.0434
8	0.0270	0.0352
9	0.0209	0.0272
10	0.0170	0.0225

**Table 5:** Number of Failures in 500 Trials with Respect to Number of Snapshots. SNR = 5 dB, Two emitters at 60° and 63°.

Snapshots	MUSIC	L1-SVD	Proposed Method
40	362	17	0
50	288	6	0
60	219	0	0
70	152	0	0
80	105	0	0
90	57	0	0
100	41	0	0
110	23	0	0
120	12	0	0
130	8	0	0
140	5	0	0
150	3	0	0
160	3	0	0
170	2	0	0
180	0	0	0
190	0	0	0
200	0	0	0

**Table 6:** Average $\beta_{0}$ and selected β value over 500 Trials with Respect to Number of Snapshots. SNR = 5 dB, Two emitters at 60° and 63°.

Snapshots	$\beta_{0}$	β
40	0.0531	0.0690
50	0.0595	0.0772
60	0.0528	0.0688
70	0.0566	0.0740
80	0.0534	0.0692
90	0.0563	0.0729
100	0.0518	0.0674
110	0.0569	0.0745
120	0.0536	0.0694
130	0.0544	0.0704
140	0.0533	0.0690
150	0.0557	0.0725
160	0.0529	0.0686
170	0.0540	0.0702
180	0.0528	0.0685
190	0.0551	0.0717
200	0.0531	0.0689

## Data Availability

Data sharing not applicable. All data generated in this paper was done through simulation and can be recreated by following the methodology presented and using the parameters we have provided.
